# Outcomes of Selective Removals for Control of Pneumonia in a Bighorn Sheep Metapopulation

**DOI:** 10.1002/ece3.70869

**Published:** 2025-05-13

**Authors:** E. Frances Cassirer, Thomas E. Besser

**Affiliations:** ^1^ Idaho Department of Fish and Game Lewiston Idaho USA; ^2^ Department of Veterinary Microbiology and Pathology Washington State University Pullman Washington USA

**Keywords:** culling, disease persistence, immunity, juvenile survival, metapopulation, wild sheep, wildlife disease

## Abstract

Pneumonia is a pervasive, population‐limiting disease of bighorn sheep ( *Ovis canadensis* ) with limited options for management. We conducted a selective removal experiment in two regions (north and south) of the Hells Canyon bighorn sheep metapopulation to test the hypothesis that pneumonia is maintained in bighorn sheep populations by chronic carriers of the bacterium *Mycoplasma ovipneumoniae* . We detected *M. ovipneumoniae* in 83 adults over 11 years across seven study populations. We removed five carriers of *M. ovipneumoniae* and nine non‐carriers from two treatment populations in northern Hells Canyon and 15 chronic carriers from a treatment population in southern Hells Canyon. We did not remove any sheep from four control populations. Local elimination of *M. ovipneumoniae* in the two northern treatment populations within a year after removals was indicated by no further detection of the pathogen, waning antibody levels, and lack of antibody in animals born after removals. Elimination in treatment populations was followed by fadeout of *M. ovipneumoniae* in the four adjacent control populations over the next 4 years without any further removals. Selective removals were associated with a decline in prevalence but did not eliminate *M. ovipneumoniae* in the southern treatment population. Clearance of infection led to nearly doubling of survival over the first 4 months of life, a 74% increase in recruitment to 7–10 months of age, and an increase in the average annual rate of population growth from 1% to 12%. The results of this experiment provide support for a focus on carriers of *M. ovipneumoniae* for mitigating low lamb recruitment associated with pneumonia‐induced mortality observed in many bighorn sheep populations across North America. However, mixed outcomes indicate that a better understanding of infection persistence and fadeout could increase the effectiveness of management interventions.

## Graphical abstract

We conducted a selective removal experiment to test the hypothesis that pneumonia is maintained in bighorn sheep populations by chronic carriers of the bacterium *Mycoplasma ovipneumoniae* . Elimination of *M. ovipneumoniae* in two of three treatment populations following removals led to significant increases in juvenile survival and population growth and was followed by pathogen clearance in adjacent populations. Removals in the third treatment population reduced prevalence but did not eliminate *M. ovipneumoniae* or result in a change in vital rates.

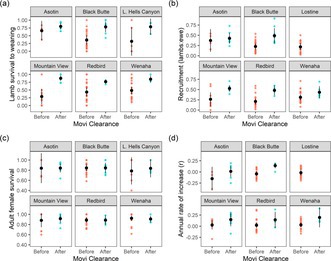

## 1 Introduction

Most epidemic diseases in humans and other animals emerge from the spillover of pathogens across species. Management strategies to limit the health effects of pathogen spillover and subsequent persistence in recipient populations have included various actions aimed at decreasing transmission or boosting host immunity. In wildlife, management often involves nonselective culling, usually with the objective of reducing the risk of pathogen transmission to domestic animals or humans. However, recently there has been an increasing emphasis on devising selective culling or removal strategies intended specifically for controlling untreatable emerging diseases threatening wildlife populations (Beeton and McCallum 2011; Wolfe et al. 2018; Miguel et al. 2020).

Pneumonia has had significant impacts on the abundance of bighorn sheep ( *Ovis canadensis* ) since the arrival of European livestock into the range of wild sheep in western North America (Cassirer et al. 2018). This polymicrobial disease typically originates from spillover of the pathogen *Mycoplasma ovipneumoniae* , a bacterium commonly found in domestic sheep ( *Ovis aries* ) and goats (*Capra aegagrus hircus*, Besser et al. 2008; Kamath et al. 2019). Disease burden in domestic sheep and goats is largely restricted to juveniles, but, although effects vary (e.g., Besser et al. 2021; Johnson et al. 2022), exposure of bighorn sheep is often associated with high rates of morbidity and mortality in all age classes (Rifatbegović, Maksimović, and Hulaj 2011; Cassirer et al. 2018; Manlove et al. 2019). Most wild sheep that survive initial exposure clear *M. ovipneumoniae* and recover completely. However, some individuals may become carriers capable of maintaining infection within populations (Plowright et al. 2017). Longer‐term demographic impacts on populations also vary, but persistently infected populations of wild sheep often experience annual or periodic high‐mortality pneumonia epizootics in juveniles, which reduces recruitment and limits population recovery (Manlove et al. 2016; Cassirer et al. 2018).

Previous efforts to mitigate the impacts of pneumonia in wild sheep have included medical treatment, vaccination, mineral supplementation, nonselective removals, population eradication, range expansion, and population augmentation (Cassirer et al. 2001; Goldstein et al. 2005; Sirochman et al. 2012; Bernatowicz et al. 2016). These strategies have produced few clear successes, and, in some cases, have been counterproductive (Cassirer and Sinclair 2007; Plowright et al. 2013; Almberg et al. 2022). However, a study conducted concurrently with this one showed that selective removal of chronic carriers of *M. ovipneumoniae* was associated with pathogen clearance and elimination of pneumonia in a population of bighorn sheep in Custer State Park, South Dakota (Garwood et al. 2020). The objectives of our study were to further evaluate the effectiveness of using selective removals of chronic carriers to eliminate *M. ovipneumoniae* and to assess the effects of clearance on population health and vital rates in the Hells Canyon metapopulation.

## 2 Materials and Methods

### 2.1 Study Area

**FIGURE 1:**
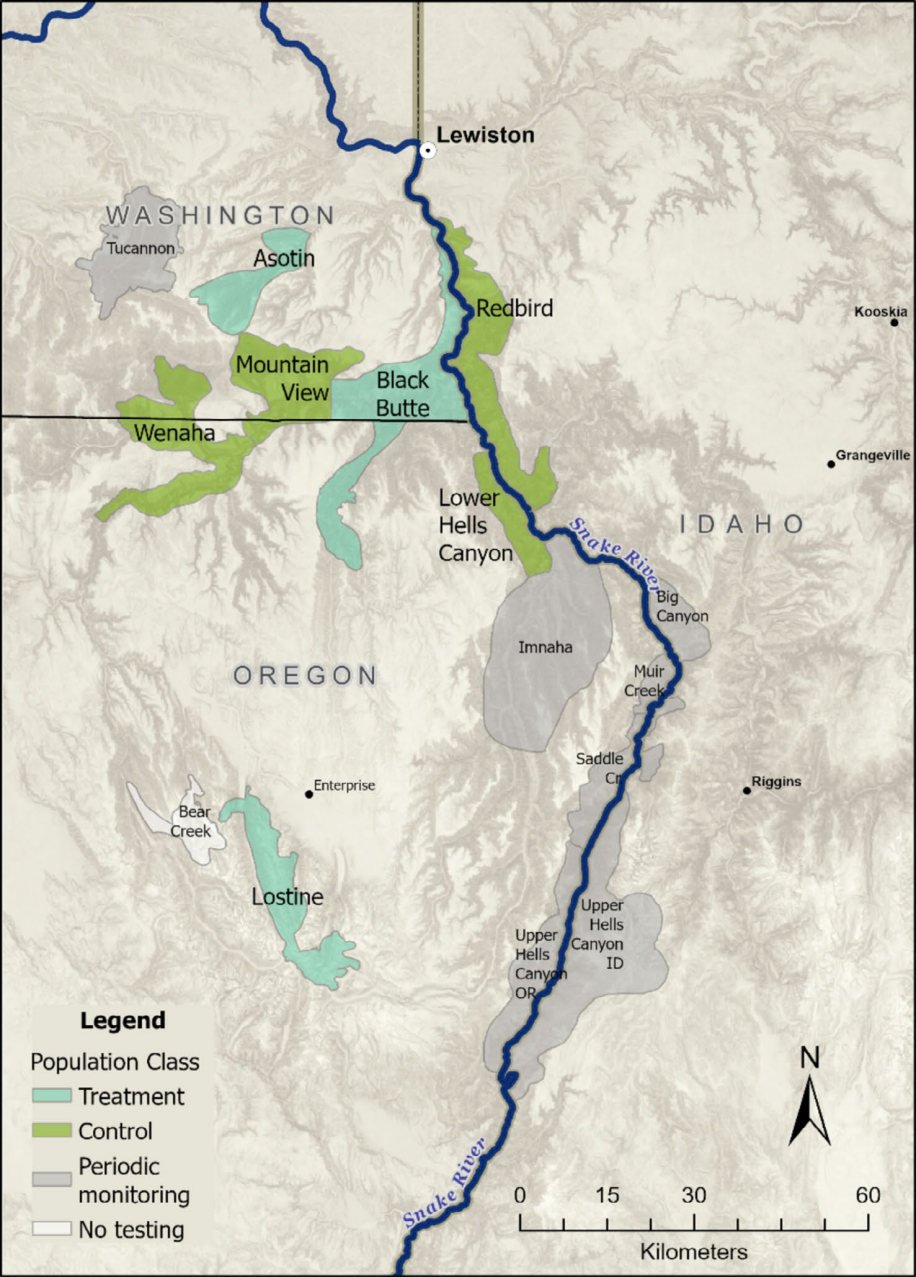
The Hells Canyon bighorn sheep metapopulation in Idaho, Oregon, and Washington surrounding the Snake River. Treatment populations where removals were conducted are shown in blue, and populations monitored as controls are in green. Populations shaded in gray were sampled periodically but were not a focus for the removal experiment.

The Hells Canyon bighorn sheep metapopulation inhabits a range of approximately 22,735 km^2^ in the Columbia Basin and Blue Mountain ecoregions of the states of Idaho, Oregon, and Washington, USA, within the traditional lands of the Nez Perce Tribe and the Confederated Tribes of the Umatilla Indian Reservation (Figure 1). Bighorn sheep are native to this area but were extirpated by 1945, likely by a combination of exposure to pathogens carried by domestic sheep, competition with domestic sheep for forage, and unregulated hunting. Reintroductions began in 1971 and were responsible for the reestablishment of 16 populations containing an estimated 800 bighorn sheep at the start of this study in 2013. Despite translocation successes, the metapopulation has a history of pneumonia outbreaks and persistent disease, which have been linked to introductions and subsequent spread of *M. ovipneumoniae* (Cassirer et al. 2013, 2017).

In 1995, an all‐age pneumonia outbreak in the Black Butte, Mountain View, Lower Hells Canyon, Wenaha, and Redbird populations in the northern region of the metapopulation was associated with the introduction of a strain of *M. ovipneumoniae* that spread south to nearly all populations between 1997 and 2011 (Figure 1, Cassirer et al. 2017). In the winter of 2011–2012, the invasion of this strain into the formerly uninfected Asotin population was accompanied by a pneumonia epidemic and a population decline of 35% to approximately 70 sheep (Bernatowicz et al. 2016). In 2013, we initiated this experiment to test whether removal of chronically infected females (chronic carriers) would clear *M. ovipneumoniae* in the Asotin population. We based the experiment on early results of intensive testing started in 2012 in the Lostine population in the Wallowa Mountains of southern Hells Canyon (*n* = 75 sheep) (Figure 1) that suggested that chronic infections were limited to a small proportion of individuals (Plowright et al. 2017). We initially focused testing on adult females because it was more logistically feasible to test the hypothesis that chronically infected females were the source of infection that led to pneumonia‐induced mortality in lambs than it was to try to test the broader hypothesis that chronic carriers (perhaps both male and female) maintained infection throughout the population. In 2014, detection of a new strain of *M. ovipneumoniae* and an associated pneumonia outbreak occurred in the Black Butte population, accompanied by a 20% population decline from approximately 50 to 40 sheep (Cassirer et al. 2017). Due to concerns about onward spread of this strain across the metapopulation, Black Butte was added as a treatment population. After the study in the Lostine was completed, we added it as another treatment population and removed the remaining females identified as chronic carriers from 2017 and 2020. The persistently infected Mountain View (*n* = 40 sheep), Wenaha (*n* = 85 sheep), Lower Hells Canyon (*n* = 45 sheep), and Redbird (*n* = 100 sheep) populations were monitored throughout the study as controls. Together, these three treatment and four control populations, containing 455 sheep, comprised the focal study areas for this research (Figure 1).

### 2.2 Capture and Health Sampling

Since 1997, we have individually marked, radio‐collared, and collected health samples from bighorn sheep in Hells Canyon. Starting in 2007, following the identification of *M. ovipneumoniae* as a key component of epidemic respiratory disease, we have submitted nasal swabs to the Washington Animal Disease and Diagnostic Laboratory (WADDL) and the Besser lab at Washington State University for detection of *M. ovipneumoniae* by polymerase chain reaction (PCR, Besser et al. 2008; Ziegler et al. 2014; Manlove et al. 2019). Strains were genotyped using multilocus sequence typing as previously described (Cassirer et al. 2017). Blood serum was analyzed with a competitive ELISA test for detection of antibody to *M. ovipneumoniae* at WADDL.

We classified population‐years as “detected” when *M. ovipneumoniae* was detected by PCR in one or more individuals at capture or mortality. Population‐years where *M. ovipneumoniae* was not detected on PCR in captures or mortalities were classified as “Not detected.” Population‐years where no samples from captures were available and no *M. ovipneumoniae* were detected on mortalities were not classified.

Estimates of *M. ovipneumoniae* infection and antibody prevalence were based on testing a cross‐section of a population within a year. In all analyses, each cross‐section included at least 4 samples per age, sex, or population (median = 15). Years refer to bighorn sheep biological years in Hells Canyon beginning on May 1 with the seasonal birth pulse and ending the following April. To avoid biasing prevalence estimates towards animals sampled more than once during a biological year, we averaged infection and exposure status for individuals within years (n positive tests/total tests).

Starting in 2012, we increased captures in treatment populations to sample as many adult females as logistically and safely possible and resampled those that tested positive for *M. ovipneumoniae* on nasal swabs to identify chronic and intermittent carriers. We classified adult females that tested positive twice over at least two consecutive years as chronic carriers (Plowright et al. 2017) and removed them from the population. In addition to adult females, we also captured and sampled a smaller number of males, yearlings, and 6‐ to 9‐month‐old lambs and tested them repeatedly where possible. Captures and sampling for follow‐up testing in treatment and control populations were conducted through 2022. All captures and handling were conducted in compliance with Idaho, Oregon, and Washington wildlife agency safety and animal welfare protocols.

### 2.3 Population Monitoring

In the northern study populations, except for Lower Hells Canyon, we observed individually marked females approximately twice a month and males once a month from the ground or from the air. The Lower Hells Canyon population was monitored less consistently because of limited access and lower priority for wildlife managers. Data recorded at each observation included the identity of marked individuals, group size and composition, lamb status of marked females (whether they had a lamb or not), and clinical signs of respiratory disease. Lambs of marked females in all northern populations, including Lower Hells Canyon, were monitored more frequently from parturition to death or October (approximately weaning). In the Lostine population, monitoring was conducted by ground and aerial telemetry observations monthly or bimonthly on remote alpine summer range between May and September and monthly following arrival on the winter range in November.

Radio collars were equipped with a motion‐sensitive switch that was activated when no movement was detected for four or more hours, indicating a possible mortality. We investigated all mortalities of marked and unmarked animals found dead in the field and brought out the entire sheep for necropsy at WADDL or conducted field necropsies. Cadavers and/or tissue and organ samples were submitted to WADDL for necropsy, histopathology, and laboratory testing.

In winter or late spring of every year, we surveyed most populations to obtain abundance and composition estimates using ground‐based mark‐resight surveys (Shenk and White 2001), aerial sightability surveys (Fieberg 2012), or minimum counts, depending on the population.

### 2.4 Data Analysis

Statistical analyses and modeling were conducted in R (RCoreTeam 2021). Annual adult female survival was estimated before and after clearance of *M. ovipneumoniae* using staggered‐entry Kaplan–Meier analysis in the Survival package (Pollock et al. 1989; Therneau 2022). We used logistic regression (general linear model with a binary outcome variable, a logit link, and a binomial error distribution) to compare the prevalence of infection and antibody among sex and age classes and populations and to compare average annual survival rates before and after the clearance of *M. ovipneumoniae* . We used a general linear model to compare population growth (ln [n year_2_/n year_1_]) before and after clearance of *M. ovipneumoniae*. We used the emmeans package (Lenth 2024) to estimate odds ratios (OR) and to conduct pairwise comparisons of marginal means using the Tukey method. Antibody decay following clearance of *M. ovipneumoniae* was modeled in individuals that were alive before and after clearance with linear and logarithmic models of antibody level (% inhibition) and time since last exposure (Appendix A).

## 3 Results

### 3.1 Health Sampling

To provide an overview and timeline of infection in the Hells Canyon bighorn sheep metapopulation, we analyzed swab and serum samples collected from 850 bighorn sheep captured in 12 populations from 1999 to 2022 (80 population‐years, Table 1). To detect infection, PCR tests for *M. ovipneumoniae* were conducted on 1220 nasal swab samples collected at captures of 788 sheep from 2007 to 2022: 943 samples from adults, 140 samples from yearlings, and 147 samples from lambs. *M. ovipneumoniae* was detected by PCR in 150 sheep (19%). We also detected *M. ovipneumoniae* by PCR in 118 mortalities from 2006 to 2014, from fresh and frozen tissue samples, and in archived formalin‐fixed, paraffin‐embedded blocks from necropsy cases at WADDL from 1997 to 2004 (Cassirer et al. 2017).

**TABLE 1:** Overview of samples collected from bighorn sheep for evaluation of *Mycoplasma ovipneumoniae* infection and exposure in 12 populations throughout Hells Canyon, 1999–2022.

Population^a^	n PCR Tests (Infection)	n ELISA tests (Exposure)	n sheep tested^b^ (PCR/ELISA)	n sheep tested more than once
Treatment populations	708	703	355 (331/338)	146
Control populations	377	403	350 (322/350)	44
Focal populations total	1085	1106	705 (652/688)	190
Nonfocal populations	145	154	145 (136/144)	14
Grand total	1230	1260	850 (788/832)	204

^a^ Focal populations in the removal experiment were classified as treatment (removals) or control (no removals). Lower‐intensity periodic testing was conducted in other populations (nonfocal) not part of the removal experiment.

^b^ The number of individuals tested by PCR or ELISA differed from the total number of individual sheep tested when there were retests and when either swab or serum samples were not available for an individual.

To characterize patterns of exposure to *M. ovipneumoniae* , we tested 1260 serum samples from 832 bighorn sheep captured 1999 and 2022 for antibody to *M. ovipneumoniae* by cELISA: 973 samples from adults, 147 samples from yearlings, and 140 samples from lambs (Table 1). Antibody to *M. ovipneumoniae* was detected in 541 samples from 403 sheep (48%).

We used subsets of the above health testing data (Table 1) to classify annual *M. ovipneumoniae* infection status of populations and to investigate how infection and antibody prevalence varied by sex, age, and among populations during years when infection was detected.

### 3.2 Patterns of Infection and Exposure

**FIGURE 2:**
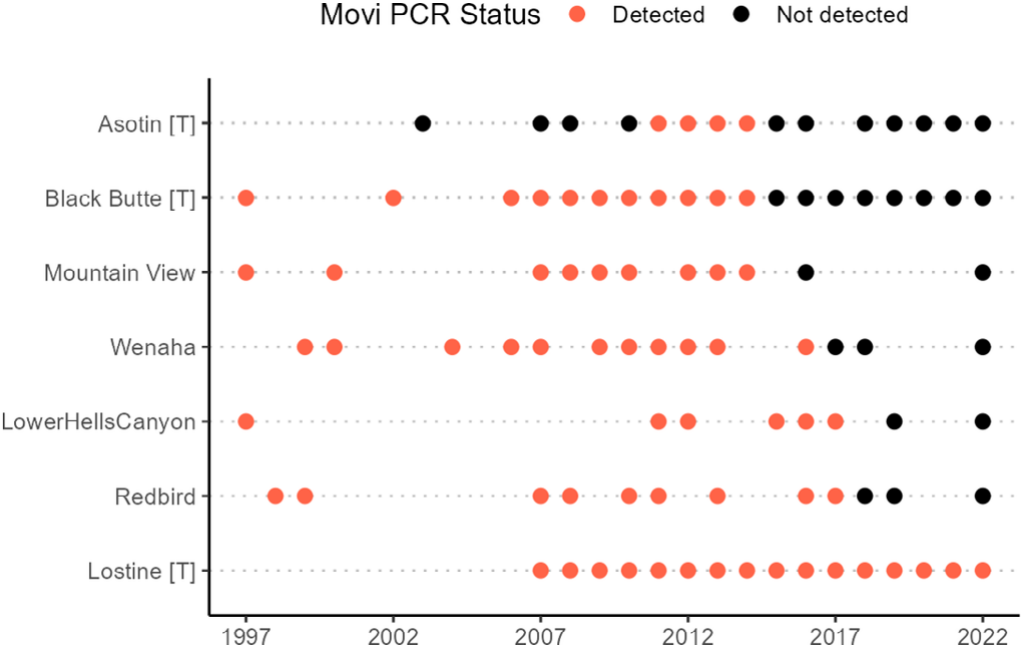
*Mycoplasma ovipneumoniae* (Movi) infection status of focal bighorn sheep populations in Hells Canyon, 1997–2022. Populations are ordered on the y‐axis generally from the northern treatment populations south. The suffix [T] indicates treatment populations where removals were conducted (Asotin, Black Butte, and Lostine).

**FIGURE 3:**
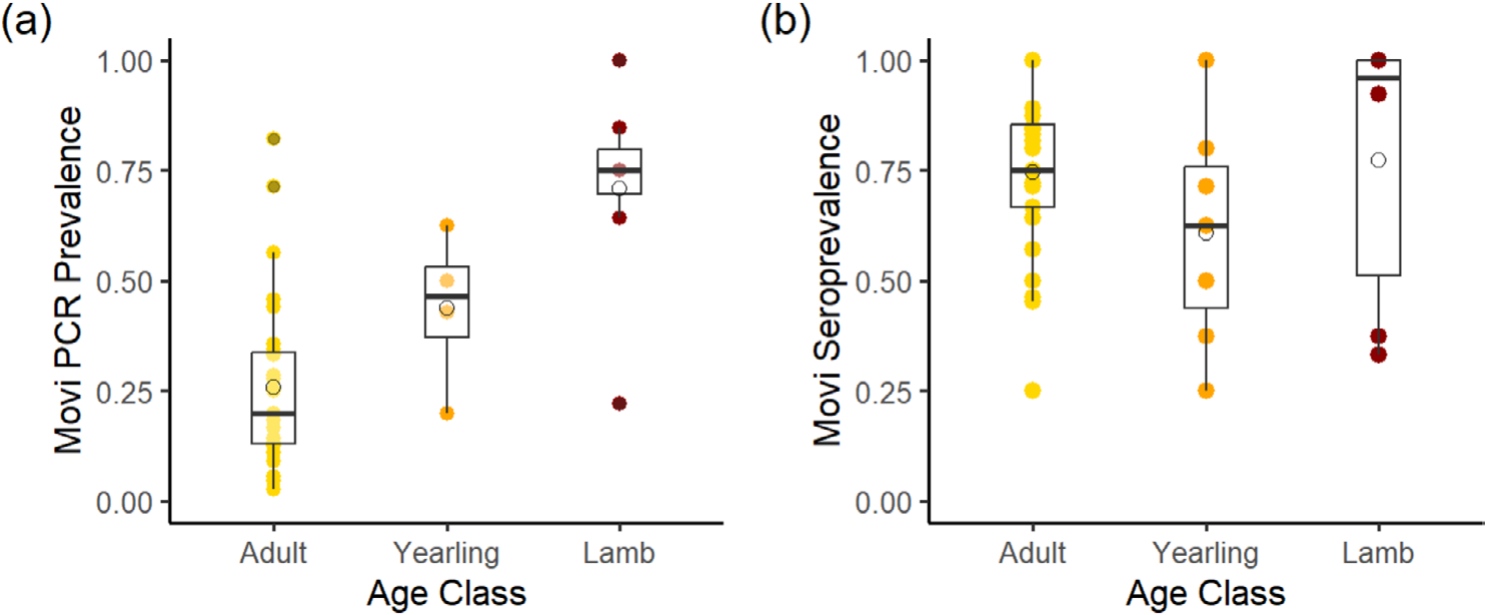
*Mycoplasma ovipneumoniae* (Movi) prevalence in adult, yearling, and juvenile bighorn sheep in infected populations in Hells Canyon, 1999–2022. Each point represents a cross‐sectional sample of a population within a biological year. (a) Proportion of nasal swab samples with detections of infection. (b) Proportion of serum samples with detectable antibody. The horizontal line in the box is the median, open circles are the mean, and the top and bottom borders are the 25% and 75% quartiles, and the whiskers extend to a distance of 1.5 times the interquartile range beyond the 25% and 75% quartiles or to the minimum or maximum value, whichever is less.

Except for the Asotin population, *M. ovipneumoniae* was detected in the seven focal study populations nearly every year from the time we routinely started testing in 2007 through at least 2014 when removals were conducted (Figure 2). Infection prevalence in lamb, yearling, and adult age classes during years when *M. ovipneumoniae* was detected was estimated from 516 nasal samples collected in 12 populations over 37 population‐years (2007–2021). Prevalence of exposure by age class was estimated from 429 serum samples collected in 12 populations over 41 population‐years from 1999 to 2021. Infection prevalence within a population‐year ranged from 0.03 to 1, differed significantly by age class, and was higher in lambs (*n* = 53, $\bar{x}$ = 0.70) than adults (*n* = 439, $\bar{x}$ = 0.27, Adult—Lamb OR = 0.165, *z* ratio = −5.710, *p* < 0.001) and intermediate in yearlings (*n* = 24, $\bar{x}$ = 0.46, Adult—Yearling OR = 0.43, Lamb—Yearling OR = 2.61, *z* ratio <1.99, *p* ≥ 0.12). Average antibody prevalence was 0.75 and did not differ among age classes (*z* ratio < 1.7, *p* > 0.22, Figure 3b). Since the only significant difference we detected among age classes was in the prevalence of *M. ovipneumoniae* infection between adults and lambs, yearlings and adults were combined into a single adult class in subsequent summaries.

There was no difference in overall infection prevalence between adult males ($\bar{x}$ = 0.21, *n* = 78, 13 population‐years) and females ($\bar{x}$ = 0.24, *n* = 452, 31 population‐ years; OR = 1.21, *z* ratio = −0.51, *p* = 0.52). Antibody prevalence was higher in adult females ($\bar{x}$ = 0.76, *n* = 458, 31 population‐years) than males ($\bar{x}$ = 0.56, *n* = 89, 15 population‐years, OR = 2.48, *Z* = 3.78, *p* < 0.001).

**FIGURE A1:**
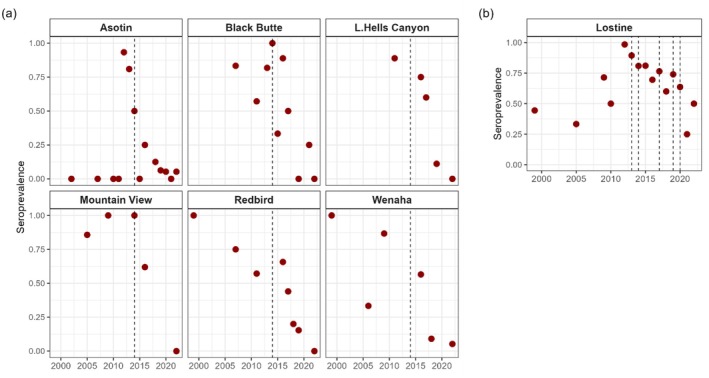
Serum antibody prevalence in seven Hells Canyon bighorn sheep populations, 1999–2022. (a) Proportion of serum samples from adults with detectable antibody 1999–2022 in six interconnected populations, where *M. ovipneumoniae* was cleared. Dashed line indicates the biological year (2014) when carriers were removed in the Asotin and Black Butte populations (main text Table 2). (b) Proportion of serum samples from adults with detectable antibody in the Lostine population, where the prevalence of *M. ovipneumoniae* infection was reduced during the study but not cleared. Dashed lines indicate years when carriers were removed (main text Table 2).

Average prevalence of infection in 541 samples from adults collected over 30 population‐years between 2009 and 2022 differed significantly among the seven focal populations and was higher in the Lostine ($\bar{x}$ = 0.39) than the other populations except Black Butte ($\bar{x}$ =0.24) and Mountain View ($\bar{x}$ = 0.22, *z* ratio >3.0, *p* ≤ 0.04). Antibody prevalence in 545 samples collected from adults over 31 population‐years between 1999 and 2021 differed across the seven focal populations and across years (Figure 3b, Figure A1). Average antibody prevalence in adults across all years (range 0.56–1) was lower in the Redbird population (0.56) than in the Black Butte (0.85) and Lostine populations (0.76, *z* ratio >3.09, *p* ≤ 0.03).

### 3.3 Testing Outcomes and Removals

During the experimental removal study, 2012–2022, we analyzed 750 nasal swab samples from 413 sheep (302 adults, 77 yearlings, and 81 lambs) collected in the focal populations during the period when *M. ovipneumoniae* was detected (exposed samples/# sheep; Asotin 89/66, Black Butte 39/29, Lower Hells Canyon 35/33, Mountain View 22/20, Redbird 108/94, Wenaha 41/41, Lostine 393/130). This included 124 adult females and 39 males tested in treatment populations, representing a sampling intensity of approximately 80%–95% of adult females, and 110 adult females and 29 males in the control populations. In the northern focal populations, *M. ovipneumoniae* was detected in 29 adults (21 F, 8 M), which represented a range in prevalence of 0.08–0.33; in the Lostine population, *M. ovipneumoniae* was detected in 54 adults (43 F, 11 M) for a prevalence of 0.55 (Table 2). Infection prevalence averaged 0.44 in yearlings (26 of 77) and 0.62 in lambs (51 of 82). Six yearlings and 30 lambs were retested at least once and up to 6 times. No lambs (*n* = 7) or yearlings (*n* = 4) that initially tested positive were positive on retest in the northern populations. In the Lostine population, 6 of 25 (24%) lambs and yearlings that initially tested positive were positive on retest. One was euthanized as a chronic carrier at age 6, and the remaining sheep, all male, either died (1), were censored (2), or current infection status is unknown (2).

**TABLE 2:** Summary of sampling, testing, and removals of bighorn sheep during years when *Mycoplasma ovipneumoniae* (Movi) was circulating in seven focal populations in Hells Canyon, 2012–2022.

Population	No. adults sampled	Sampling period	Estimated % females tested	Movi PCR Positive (%)	No. retested^a^	No. positive on retest	No. removed^b^
Asotin [T]^c^	38	2012–2014	90%	5 (13%)	3	2	8
Black Butte [T]	27	2013–2014	80%	9 (33%)	4	0	8
Lower Hells Canyon	29	2016–2017	95%	2 (7%)	0	NA	0
Mountain View	22	2014	76%	2 (9%)	1	0	0
Redbird	75	2016–2017	95%	10 (13%)	4	0	0
Wenaha	13	2016	25%	1 (18%)	1	0	0
Lostine [T]	98	2012–2022	95%	54 (55%)	52	28	15

*Note:* Sampling reported in this table represents a subset of the data in Table 1.

^a^ Adults that tested positive were not retested (*n* = 18) if they died (55%) or were removed (6%) prior to retesting, if they were censored because they were never seen again (dispersed or died, 17%), or if they could not be recaptured (22%). All sheep that could not be recaptured (3 *M*, 1 *F*) were in the control Redbird population.

^b^ In addition to *M. ovipneumoniae* carriers, 5 noncarriers were removed from Asotin and 6 noncarriers were removed from Black Butte and transferred to captive research facilities for a separate study.

^c^ [T] indicates treatment population where carriers were removed in 2014 (Asotin *n* = 3 and Black Butte *n* = 2) or 2013–2020 (Lostine *n* = 15).

*M. ovipneumoniae* was detected in 64 adult females at least once, and 30 were positive on retest (Asotin 2, Lostine 28) and classified as chronic carriers. No males were identified as chronic carriers. All chronic carriers were removed or died over the course of the study. In Asotin, we removed two chronic carrier females in 2014. We removed 15 chronic carrier females from Lostine between 2013 and 2020 (2013–3, 2014–2, 2017–7, 2020–1, 2021–2). In Asotin we also removed one carrier (tested positive once) and 2 non‐carrier males in 2014 and three non‐carrier females in 2013. In 2014, we removed 2 carriers and 6 non‐carrier females from the Black Butte population. All animals removed through 2017 were transferred to captive research facilities for a separate study. By 2021, all females that had previously tested positive in treatment populations had been removed, died, or cleared infection (Table 2).

### 3.4 Post‐Removal Health Monitoring

**FIGURE 4:**
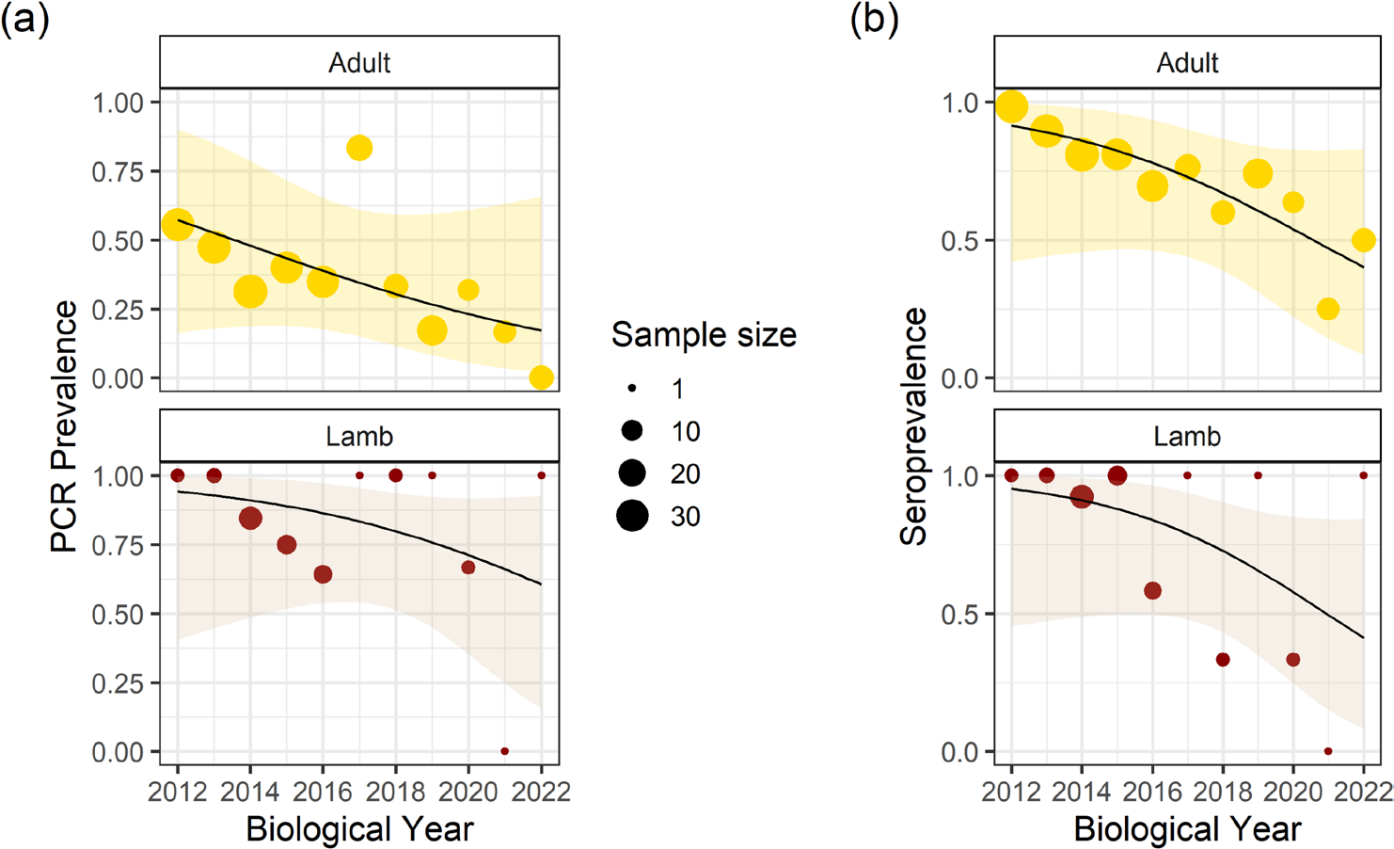
Trend in prevalence of (a) infection and (b) exposure to *Mycoplasma ovipneumoniae* in adults and lambs in the Lostine bighorn sheep population over the period of intensive testing and removal of 15 chronic carriers, 2012–2022. The black line and shaded area representthe logistic regression and associated 90% binomial confidence region.

Following removals, outcomes differed among regions. No *M. ovipneumoniae* was detected on nasal swab samples collected (Asotin *n* = 100, Black Butte *n* = 51) in the northern treatment populations through 2022, and no antibody to *M. ovipneumoniae* was detected in animals that were born after the removals (Asotin *n* = 35 samples from 27 sheep, Black Butte *n* = 14 samples from 14 sheep). In contrast, we observed significant declines in infection (*Z* = −3.67, 10 *df*, *p* < 0.001) and antibody (*Z* = −5.13, 10 *df*, *p* < 0.001) prevalence in adults in the Lostine treatment population, but *M. ovipneumoniae* was not cleared. A declining trend in infection was not significant in lambs in the Lostine population, where sample sizes were smaller (*Z* = −1.193, 10 *df*, *p* = 0.23), although antibody prevalence declined significantly (*Z* = −2.710, 10 *df*, *p* = 0.007; Figure 4).

In 2016, 2 years after the removal of carriers in Asotin and Black Butte, no *M. ovipneumoniae* was detected on nasal swab samples collected from 26 sheep in the Mountain View control population, nor was antibody detected in 5 lambs and 5 yearlings. Subsequently, no infection or antibody was detected in a previously positive adult retested in the Wenaha control population in 2017, and testing in 2018 and 2019 detected no infection and no antibody in lambs in the Wenaha, Redbird, and Lower Hells Canyon populations. No further *M. ovipneumoniae* was detected on 318 swab samples collected following fadeout through 2022 in northern treatment and control populations (Figure 2).

**FIGURE 5:**
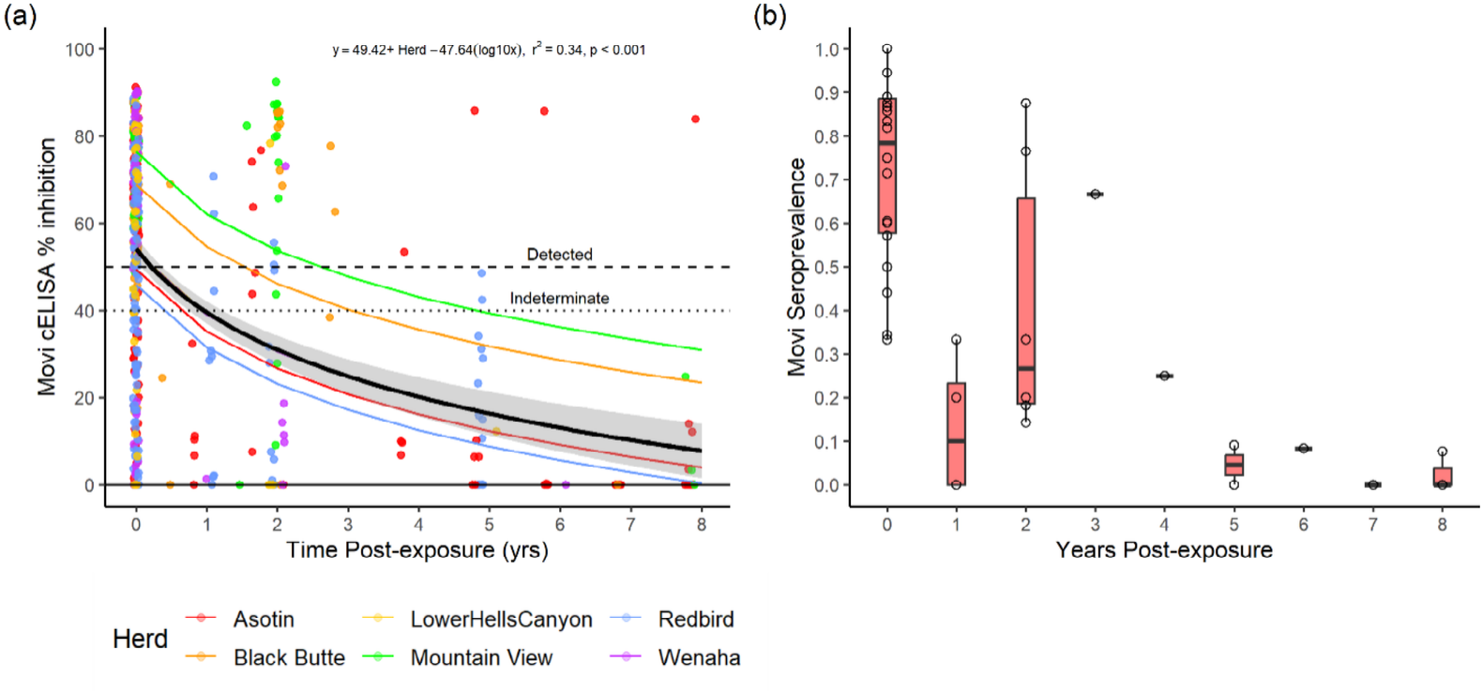
Measures of antibody to *Mycoplasma ovipneumoniae* in bighorn sheep when exposed and 1–8 years following clearance of infection in six Hells Canyon populations (herds) 1997–2022. (a) Antibody levels (cELISA % inhibition) of individual sheep during (Year 0) and in years following the last known exposure (detection of *M. ovipneumoniae* by PCR) in the population. Horizontal dashed and dotted lines represent cutoffs for classifying % inhibition scores as detected (> 50%), indeterminate (40%–50%), or not detected (< 40%). The black line and shaded area represent the overall mean and standard error of the cELISA score decay curve based on the equation at the top of the plot containing a significant additive effect for population (Herd). Thin colored lines show herd‐specific intercepts for the overall decay curve (Appendix A). (b) Prevalence of antibody detections during cross‐sectional sampling of populations when *M. ovipneumoniae* was present and in years 1–8 following the last detection. Box plots represent values as in Figure 3.

**FIGURE A2:**
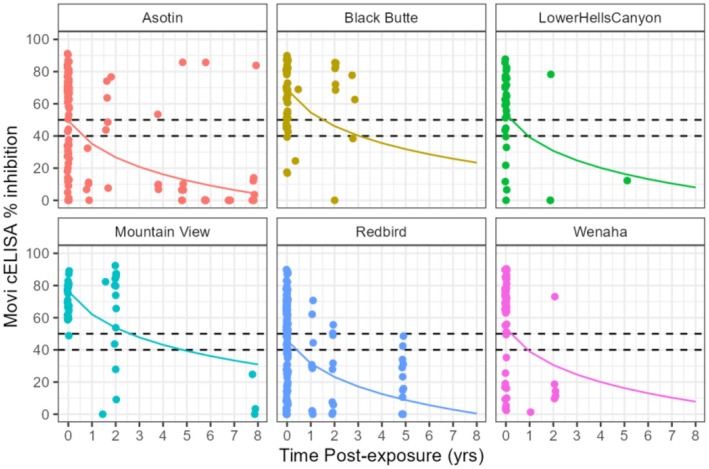
Herd‐specific patterns of *M. ovipneumoniae* antibody levels in a logarithmic decay model in Hells Canyon bighorn sheep during a selective removal study 2012–2022. Horizontal dashed lines represent cutoffs for classifying % inhibition scores as detected (>50%), indeterminate (40%–50%), or not detected (<40%).

Average antibody scores in individuals that had been exposed dropped below the cut‐off for classification as “detected” (50%) the year after the last detection of *M. ovipneumoniae* and mean cELISA scores declined from an average of 54% inhibition in exposed populations to less than 10% 5–8 years post‐exposure. Estimated antibody half‐life was about 3 years. Antibody score in 56% (25 of 45) of sheep tested from 5 to 8 years post‐exposure remained above 0 (7%–49%), and one previously exposed individual each in the Asotin and Black Butte populations continued to maintain detectable antibody up to 8 years post‐exposure. Sheep in the Black Butte and Mountain View populations had higher average cELISA scores when exposed (Black Butte 69%, Mountain View 77%) than other populations (Figure 5a, Tables A1 and A2, Figure A2). Seroprevalence reached a median close to 0 by year 5 post‐exposure (Figure 5b).

### 3.5 Demographic Response to Clearance

**FIGURE 6:**
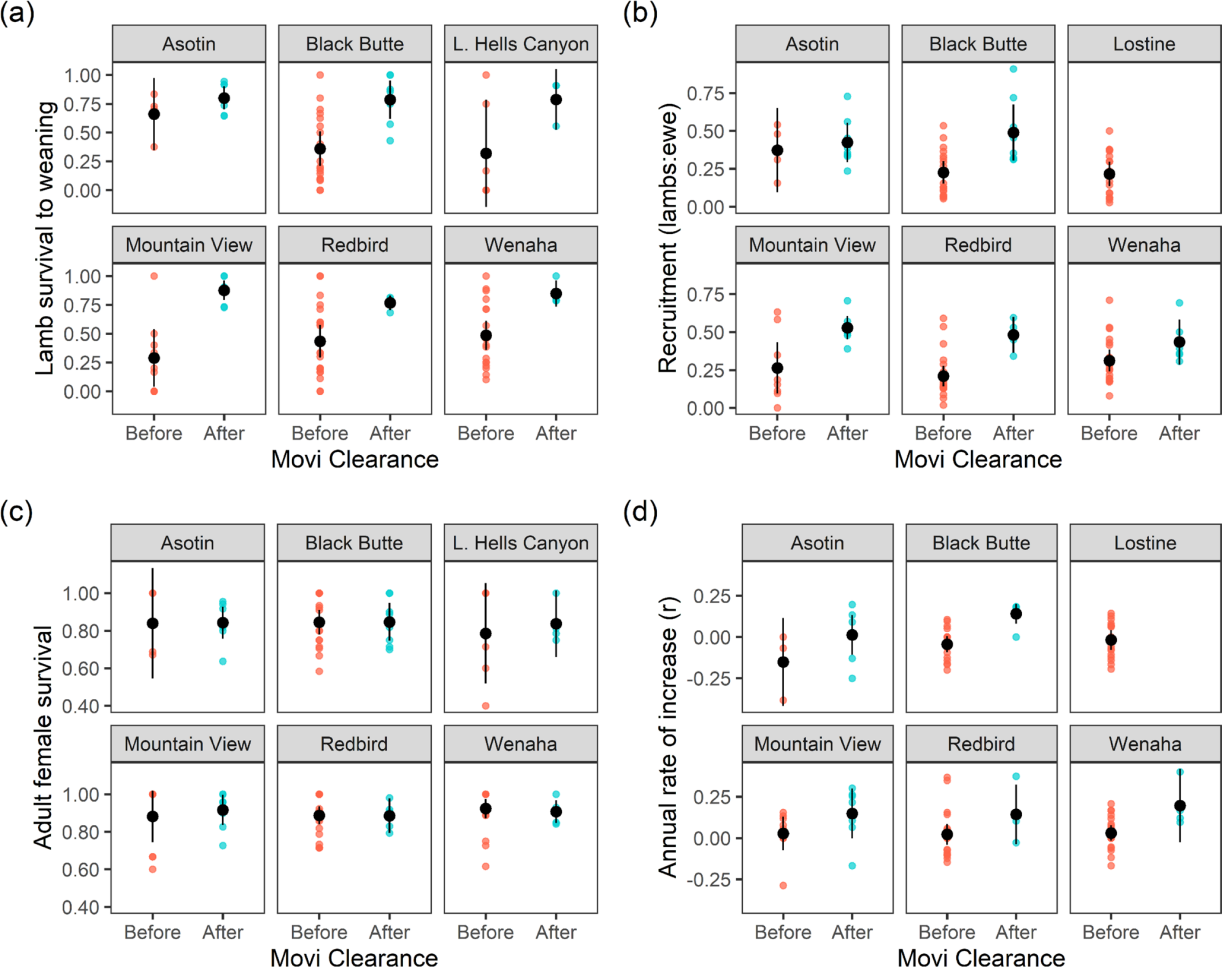
Demographic responses were observed following the clearance of *Mycoplasma ovipneumoniae* (Movi) in Hells Canyon bighorn sheep populations. Colored points represent annual estimates, and black points and error bars show the mean and 95% confidence interval. (a) Survival of lambs born to marked females to approximately 4 months of age. (b) Ratio of lambs: Female at 7–10 months of age. (c) Annual survival of adult females. (d) Annual rate of population growth (*r*). Not all demographic metrics were available for the Lower Hells Canyon and Lostine populations.

Between 1997 and 2022, we monitored the survival and productivity of 376 marked females in the six northern populations (Asotin *n* = 93, Black Butte *n* = 62, Lower Hells Canyon *n* = 23, Mountain View *n* = 40, Wenaha *n* = 56, Redbird *n* = 102) and 104 marked females in the Lostine population in southern Hells Canyon. Prior to clearance of *M. ovipneumoniae* , high mortality pneumonia outbreaks in lambs at 6–10 weeks of age were frequent; lamb survival to weaning averaged 42%, and average recruitment was estimated at 0.27 lambs: female. After *M. ovipneumoniae* clearance, no pneumonia outbreaks were observed in lambs, and survival to weaning nearly doubled to 82% (*z* ratio = 3.82, *p* = 0.001). Variation in survival to weaning was lower within and among populations in the absence of *M. ovipneumoniae* (Figure 6a). Recruitment following clearance increased 74% to 0.47 lambs: female (*z* ratio = 2.35, *p* = 0.029, Figure 6b), while there was no significant change in average annual ewe survival (88% when *M. ovipneumoniae* was present and 87% when it wasn't (*z* ratio = 0.14, *p* = 0.989, Figure 6c)). Annual population growth rate increased from an average of 0.01 to an average of 0.12 (*t*.ratio = 4.17, *p* = 0.001, Figure 6d). No change in recruitment or rate of population growth was observed when the prevalence of *M. ovipneumoniae* was reduced but not cleared in the Lostine population (recruitment 0.18 before removals and 0.23 during and after removals, *z* ratio = −0.260, *p* = 0.795; *r* = 0 before, during, and after *z* ratio −0.26, *p* = 0.795).

## 4 Discussion

We found that removals of adult carriers of *M. ovipneumoniae* were followed by local elimination of the pathogen in two persistently infected populations of bighorn sheep. In one population we removed two female chronic carriers and one male carrier not confirmed to be chronic; in the other we removed two female carriers not confirmed to be chronically infected. Following removals, there were no further detections of *M. ovipneumoniae* , no detectable antibody in animals born post‐removal, and a decline in antibody scores in previously exposed individuals. Unexpectedly, elimination of *M. ovipneumoniae* in these populations was followed by geographically sequential clearance in the four neighboring control populations over 4 years without any removals. In contrast, selective removal of female chronic carriers was associated with a decline in prevalence and exposure but not elimination of *M. ovipneumoniae* in a third treatment population.

### 4.1 Role of *M. ovipneumoniae* in Population Dynamics

Clearance of *M. ovipneumoniae* resulted in nearly doubling survival over the first 4 months of life and an increase in the average annual rate of population growth by over an order of magnitude. Annual variation in early lamb survival declined after clearance because lambs no longer experienced years with very high rates of mortality. Overall, *M. ovipneumoniae* exposure was supported as a primary factor limiting lamb survival and, in most populations, the rate of population growth. This is consistent with negative effects of *M. ovipneumoniae* infection on bighorn lamb survival reported in other studies (Butler et al. 2018; Garwood et al. 2020; Besser et al. 2021; Paterson et al. 2021).

Juveniles were the age class most susceptible to infection, and there was a high level of exposure across all ages, reflecting both the lack of effective immunity to infection in lambs and the gregarious nature of bighorn sheep, which facilitates pathogen transmission. Significant variation in infection and antibody prevalence among populations and sampling events may represent waves of infection alternating with stochastic or partial fadeouts over time and may correspond with previous observations of high annual variation in lamb survival and detection of pneumonia (Cassirer et al. 2013; Paterson et al. 2021). Failure of a carrier to produce a lamb, movement of a carrier away from the area, or death of a carrier could each reduce the likelihood of transmission to lambs, increasing survival of a lamb cohort (Besser et al. 2021; Spaan et al. 2021).

### 4.2 Factors Influencing Outcomes of Selective Removals

Selective removals for management of disease in wildlife have had mixed results, largely due to complications related to the epidemiology of the pathogen, characteristics of the host population, and availability of reliable diagnostic testing (Miguel et al. 2020). Successful clearance of *M. ovipneumoniae* following selective removals from the Asotin treatment population in this study and in a previously reported study (Garwood et al. 2020) is likely due to aspects of the disease that make it conducive to application of this management strategy. The pathogen is generally host‐specific, does not persist in the environment (Citti and Blanchard 2013; Walsh et al. 2023), and the PCR test for detecting *M. ovipneumoniae* is an accurate indication of active infection and shedding (Walsh et al. 2016). A central assumption is that long‐term infections (chronic carriage) in specific individuals are the mechanism driving the persistence of *M. ovipneumoniae* in bighorn sheep populations, a hypothesis that was supported and challenged in this study. Overall, we found that most infections were transient (< 1 year). Removal of chronically infected individuals was followed by clearance of *M. ovipneumoniae* in the Asotin population as predicted. In the Black Butte population, although we removed individuals infected with *M. ovipneumoniae* , these infections had not been documented to be chronic prior to removal. It is possible that any chronic carriers died in the pneumonia outbreak that occurred approximately 5 months prior to removals. If so, removals may have had no effect or may have prevented recruitment of new chronic carriers.

Infections and exposure were reduced in the Lostine population, but *M. ovipneumoniae* was not cleared, despite the removal of all known chronic carriers. Variation in antibody and infection prevalence among populations and the persistence of *M. ovipneumoniae* in the Lostine treatment population suggest that there are important epidemiological differences among bighorn sheep populations and over time. For example, higher antibody scores were observed in Black Butte and Mountain View compared to other populations (Figure 5, Appendix A) prior to clearance, which corresponded temporally to the boosting of antibody levels observed following the most recent invasion of *M. ovipneumoniae* and an associated pneumonia outbreak in 2014 (Cassirer et al. 2017). High infection prevalence, as observed in the Lostine population, may be associated with reduced host resistance, increased strain virulence, or higher transmission rates, which could interfere with pathogen clearance. This population is concentrated on limited winter range, likely increasing opportunities for transmission. It is also the only population where sinus tumors, abnormal masses in the sinuses that can impede clearance of bacteria (Fox et al. 2015), were detected. A previous study suggested that lower genetic diversity may be associated with chronic carriage in this population (Plowright et al. 2017; but see Martin et al. 2021). Finally, although the strain of *M. ovipneumoniae* detected in this population is a genetic match to the dominant strain in northern Hells Canyon, the ability to maintain long‐term infections could differ within strains of this genetically diverse bacterium (Citti, Nouvel, and Baranowski 2010).

One of the most surprising, confounding, and encouraging aspects of this study was the gradual clearance of infection and disease in the control populations following the elimination of *M. ovipneumoniae* in the treatment populations (Figure 2). In bighorn sheep metapopulations, populations are typically defined by distinct core ranges connected by periodic interpopulation movements (Bleich et al. 1996). Connectivity across structured populations facilitates the spread of pathogens and can sometimes allow diseases to persist when they could not in isolated populations (Hess 1996). Clearing infection within the Asotin population and deaths, removal of carriers, and depopulation of a social group in the Black Butte population may have eliminated sources of exposure to adjacent populations, thus tipping the balance towards pathogen extinction in the control populations. Movement of marked animals and spread of *M. ovipneumoniae* strains among populations was documented during and prior to the study (Cassirer et al. 2017). However, without knowing global transmission rates among populations before and after removals and their contribution to pathogen persistence, we cannot conclusively attribute fadeouts in the control populations to removals in the treatment populations.

### 4.3 Future Direction

This work highlights potential areas for future research on factors that might influence efforts to promote pathogen clearance in bighorn sheep and other species, including improving our understanding of carriage and the chronic carrier state, the role of males in pathogen persistence and spread, and identifying causes of observed variation in infection and exposure prevalence within and between populations. This research would benefit from cross‐disciplinary collaboration between field studies, incorporating data from this and other ongoing and completed studies to clear infection from bighorn sheep populations through selective removals, laboratory investigations to investigate characteristics influencing host immunity and pathogen virulence, and contributions from iterative disease modeling. Connectivity and social organization play a fundamental role in the dynamics of infectious disease and together can promote pathogen persistence or extinction (Sah et al. 2017). Developing network‐based disease dynamic models (Silk et al. 2019) and building on existing dynamic compartmental models of disease management in wild sheep (Almberg et al. 2022) could shed light on the results of this experiment and provide an opportunity to develop and test hypotheses about factors influencing outcomes of management interventions as well as contributing to conceptual models for managing other persistent diseases.

## 5 Conclusions

The results of this research reinforce the importance of *M. ovipneumoniae* as a focus for management of pneumonia in bighorn sheep. Selective removal of chronic carriers can be an effective method of clearing or reducing infection in populations, but pathogen extinction may be more likely when there are few carriers and lower rates of transmission (Almberg et al. 2022). In addition, this study suggests that selective removals of carriers might lead to the fadeout of pneumonia in bighorn sheep metapopulations by disrupting the longer‐term spatiotemporal dynamics of infection within and among populations. Annual dynamics of disease and infection appear to be asynchronous between adjacent populations (Cassirer et al. 2013), and intra‐ and inter‐population connectivity and transmission rates may influence whether or not infection persists. Further investigation is needed to better understand the mechanisms driving *M. ovipneumoniae* fadeout and persistence within individuals, populations, and across metapopulations.

## Author Contributions

**E. Frances Cassirer:** conceptualization (equal), data curation (lead), formal analysis (lead), funding acquisition (lead), investigation (equal), methodology (equal), project administration (lead), writing – original draft (lead), writing – review and editing (equal). **Thomas E. Besser:** conceptualization (equal), data curation (supporting), investigation (equal), methodology (equal), writing – original draft (supporting), writing – review and editing (equal).

## Conflicts of Interest

The authors declare no conflicts of interest.

## Data Availability

Data are available on Dryad Digital Repository: https://datadryad.org/stash/share/Ebur60zoYq6tBPpt‐b8J8Tg‐f8lyY8ZdEcmP9OneiOY and will be made public upon acceptance for publication.

## APPENDIX A

Spatiotemporal patterns of *Mycoplasma ovipneumoniae* antibody prevalence and models of antibody decay in the Hells Canyon bighorn sheep metapopulation.

*Mycoplasma Ovipneumoniae* serum antibody prevalence in Hells Canyon varied across bighorn sheep populations and fluctuated over time, ranging from 0 to 1, reflecting exposure status. Seroprevalence declined across all populations during the study (2013–2022, Figure A1).

Models of antibody decay.

To investigate the pattern of antibody decay following clearance of infection, we analyzed serum antibody levels (% inhibition) in 349 samples collected in six populations during years when *M. ovipneumoniae* was detected and 153 samples collected from 1 to 8 years after the last detection (post‐exposure) by sampling and resampling sheep known to be alive when *M. ovipneumoniae* was present based on their age and capture history. We used Akaike's information criterion corrected for small sample sizes (AICc, Akaike 1973; Burnham and Anderson 2002; Mazerolle 2023) to compare six candidate models of antibody decay incorporating two parameters: Time since exposure and population (herd, Table A1). All models were statistically significant (*p* < 0.001).

**TABLE A1:** Models compared for *Mycoplasma ovipneumoniae* antibody decay in Hells Canyon bighorn sheep populations listed in descending order of fit.^a^

Model	*K*	AIC_c_	Delta AIC_c_	AIC_c_ weight	Log‐likelihood
Log_10_Yrs post‐exposure + herd	8	4793.25	0	0.78	−2388.48
Log_10_Yrs post‐exposure _*_ herd	13	4796.93	3.69	0.12	−2385.1
Yrs post‐exposure + herd	8	4797.81	4.57	0.08	−2390.76
Yrs post‐exposure _*_ herd	13	4800.89	7.65	0.02	−2387.08
Years post‐exposure	3	4852.25	59.01	0	−2423.10
Log_10_Years post‐exposure	3	4854.31	61.07	0	−2424.13

^a^ AICc is computed as (−2 * log − likelihood +2 * K * (*n*/(*n* − K − 1))). K corresponds to the number of estimated parameters, and *n* is the sample size. Delta AIC_c_ is the difference in AIC_c_ between each model and the top model in the set being compared. AIC_c_ weight quantifies the relative likelihood of each model being the best fit to the data. In this comparison, models including herd as a covariate contain 100% of the AIC_c_ weight.

Inclusion of herd greatly improved the fit of regression models, and a logarithmic rate of decay was a better fit than a linear decay rate. The top model included log time since exposure and an additive effect for herd (Table A1). In the top model, bighorn sheep in two populations, Black Butte and Mountain View, had significantly higher average *M. ovipneumoniae* antibody levels (intercepts) prior to clearance (Table A2, Figure A2).

**TABLE A2:** Coefficients for an *Mycoplasma ovipneumoniae* antibody decay model (*R* ^2^ = 0.34, *F*_6,505 *df*_ = 42.87) incorporating time since exposure with an additive effect for population in samples from six Hells Canyon bighorn sheep populations.

	Estimate	Std. Error	*t* value	Pr(>|t|)	Signif. code^a^
(Intercept)	49.42	2.498	19.787	<0.001	***
Log_10_(yrs_post_exp)	−47.64	3.79	−12.56	<0.001	***
Black Butte	19.44	4.084	4.761	<0.001	***
Lower Hells Canyon	4.03	4.679	0.861	0.389	
Mountain View	26.97	4.295	6.28	<0.001	***
Redbird	−3.554	3.079	−1.154	0.249	
Wenaha	3.86	3.955	0.976	0.33	

^a^ Significance code *** *p* < 0.001
